# The Chemotherapeutic Agent DMXAA as a Unique IRF3-Dependent Type-2 Vaccine Adjuvant

**DOI:** 10.1371/journal.pone.0060038

**Published:** 2013-03-21

**Authors:** Choon Kit Tang, Taiki Aoshi, Nao Jounai, Junichi Ito, Keiichi Ohata, Kouji Kobiyama, Benoit H. Dessailly, Etsushi Kuroda, Shizuo Akira, Kenji Mizuguchi, Cevayir Coban, Ken J. Ishii

**Affiliations:** 1 Singapore Immunology Network, Agency for Science and Technology Research, Singapore; 2 Malaria Immunology Laboratory, Immunology Frontier Research Centre (IFReC), Osaka University, Japan; 3 Host Defense Laboratory, IFReC, Osaka University, Japan; 4 Vaccine Science Laboratory, IFReC, Osaka University, Japan; 5 Laboratory of Adjuvant Innovation, NIBIO, Osaka, Japan; 6 Laboratory of Bioinformatics, National Institute of Biomedical Innovation (NIBIO), Osaka, Japan; Duke-National University of Singapore Graduate Medical School, Singapore

## Abstract

5,6-Dimethylxanthenone-4-acetic acid (DMXAA), a potent type I interferon (IFN) inducer, was evaluated as a chemotherapeutic agent in mouse cancer models and proved to be well tolerated in human cancer clinical trials. Despite its multiple biological functions, DMXAA has not been fully characterized for the potential application as a vaccine adjuvant. In this report, we show that DMXAA does act as an adjuvant due to its unique property as a soluble innate immune activator. Using OVA as a model antigen, DMXAA was demonstrated to improve on the antigen specific immune responses and induce a preferential Th2 (Type-2) response. The adjuvant effect was directly dependent on the IRF3-mediated production of type-I-interferon, but not IL-33. DMXAA could also enhance the immunogenicity of influenza split vaccine which led to significant increase in protective responses against live influenza virus challenge in mice compared to split vaccine alone. We propose that DMXAA can be used as an adjuvant that targets a specific innate immune signaling pathway via IRF3 for potential applications including vaccines against influenza which requires a high safety profile.

## Introduction

DMXAA was developed as a vascular disruptive agent for use in cancer therapy. Several clinical trials, including a recently completed phase III clinical trial for non-small cell lung carcinoma, have shown that DMXAA is safe and well-tolerated in humans [1]. It is a cell-permeable small molecule which reduces tumor load by inducing apoptosis in tumor vascular endothelium thereby reducing blood flow to solid tumor [2]. Further investigations into the properties of DMXAA have revealed that it is a strongly immunogenic molecule. The anti-neoplastic property of DMXAA is largely attributed to its induction of TNFα which can be detected in the serum and tumor micro-environment within hours of administration [3]. It can activate several inflammatory cell signaling pathways, including extracellular signal-regulated kinases 1 and 2, c-Jun N-terminal kinases, and cytosolic nucleotide-binding oligomerization domain 1 and 2-like receptors [4], [5]. In addition, DMXAA is a strong inducer of reactive oxygen species (ROS) [6]. The most striking immunogenic feature of DMXAA is its induction of immediate and predominant type-I-IFN [7]. DMXAA resembles viral infections and double stranded DNA (dsDNA) in the inflammatory signaling events it triggers to induce type-I-IFN production [8]. It utilizes the TBK1-IRF3 signaling pathway without the involvement of Toll-like receptors (TLRs) or RNA helicases for its mechanism of type-I-IFN induction. For the cell signaling events that are upstream of TBK1 phosphorylation, DMXAA was shown to initiate the translocation of the E3 ubiquitin ligase tripartite motif 56 (TRIM56) from the cytoplasm into intracellular punctate structures where the Stimulator of Interferon Genes (STING) was simultaneously recruited [9]. STING is an adaptor molecule that is vital to the induction of type-I-IFN during viral infection [10] and stimulation with cytosolic dsDNA [11] and the bacterial second messenger product, cyclic diguanylate (c-di-GMP) [12]. DMXAA was recently demonstrated to require STING for the production of IFN-β [13]. Due to its ability to induce strong type-I-IFN, DMXAA was found to be an effective antiviral agent against influenza [14], [15].

In addition to the induction of pro-inflammatory cytokines, DMXAA can induce the direct activation of antigen presenting cells (APCs) such as macrophages and dendritic cells (DCs). *In-vivo* administration of DMXAA induced maturation of DCs in draining lymph node of tumor bearing mice within 24 h. This was shortly followed by the increase of tumor antigen specific CD8 T cells and their migration to tumor sites due to chemokines such as CCL2 and CXCL10 that were released by the activated APCs [16]. Based on these immunogenic properties of DMXAA, we hypothesize that DMXAA could function as an adjuvant. In this report, we demonstrate in mouse models that DMXAA could indeed promote the adaptive immune response in immunization studies against influenza virus and be a potential adjuvant candidate.

## Materials and Methods

### Mice and immunizations

*Ifnar^-/-^* and *Irf3^-/-^* mice were of C57BL/6 background and *IL-33^-/-^* mice were of BALB/c background. The development of these animals was described elsewhere [17]–[19]. Wild-type (WT) controls were purchased from CLEA, Japan. All animal experiments were conducted in accordance with the guidelines of the Animal Care and Use Committee of Research Institute for Microbial Diseases and Immunology Frontier Research Center of Osaka University, who specifically approved this study. All animal experiments were performed to ameliorate suffering according to the guideline of ASUDC of RIMD and IFREC of Osaka university. Endotoxin-free chicken egg Ovalbumin (OVA) (Seikagaku Biobusiness) was mixed with various adjuvants, including DMXAA (Sigma-Aldrich), aluminum hydroxide suspension (Sigma Aldrich) and K-type CpG ODN 2006 (InvivoGen), in PBS prior to immunization. DMXAA was dissolved in 5% NaHCO_3_ and was ensured endotoxin-free by analysis with LAL testing (Lonza). In all immunization experiments, mice were injected intradermally at the base of tail on days 0 and 14 and were bled on day 21.

### Generation and *in-vitro* stimulation of bone marrow derived dendritic cells

*In-vitro* grown DCs were prepared by incubating red blood cells-lysed bone marrow cells from WT and various knockout mice with 20 ng/ml of GM-CSF (Peprotech, NJ, USA) for 5 days as previously described in [20]. On day 5, DCs were stimulated with DMXAA, lipopolysaccharides (LPS) (Sigma Aldrich, MO, USA), and Lipofectamine 2000 (Invitrogen, NY, USA) complexed c-di-GMP (Biolog, Bremen, Germany) for 6 h before the supernatant were collected and cytokines measured. The level of DC maturation induced by the various stimuli were determined by using flow cytometry to detect CD86 expression on CD11c^+^ cells and presented as histogram plots.

### Cytokine ELISA

TNFα was measured using the R&D DuoSet® ELISA Development Systems (R&D Systems). IFNβ was measured by ELISA, using rat monoclonal [7F–D3] antibody to Interferon beta (ab24324, Abcam) and rabbit polyclonal antibody to Interferon β (#AB2215, Millipore) and finally with sheep antibody to rabbit IgG (H&L-HRP; ab97095, Abcam). Standard curves were generated using recombinant mouse IFNβ (12400-1, Interferon Source PBL). Results reported in the figures are averages of three samples with errors displayed as standard deviations. Antibody responses to OVA and SV were determined by ELISA where plates were coated with OVA protein and SV respectively. The OVA and SV specific antibodies were detected using goat anti-mouse IgG, IgG1, IgG2a or IgG2c-HRP (Southern Biotech). The relative antibody titers were determined directly from the standard curve generated from positive serum by solving the regression line equation. All ELISAs were developed with the KPL TMB Microwell Peroxidase Substrate System (KPL).

### Influenza virus infection and vaccination

Mice were immunized intradermally, at the base of the tail, on days 0 and 14, with 100 µg DMXAA and 0.75 µg of New Caledonia/20/1999 (H1N1), prepared as described [21]. On day 21, the immunized mice were anesthetized with ketamine before they were intranasally infected with 1×10^5^ pfu of A/Puerto Rico/8/34 (PR) (H1N1) virus. All efforts were made to reduce suffering to the animal. Challenged mice were monitored daily for their body weight loss and any signs of sickness. Mice that were in a moribund condition or had loss more than 25% of body weight were considered to have reached an experimental endpoint and were humanely euthanized by cervical dislocation.

### Statistical analysis

All data were reported as means ± standard deviation. Students t-test was used to compare significant differences between two groups, whereas one-way analysis of variance with Bonferroni's post-test was used to compare differences among three or more groups. Log-rank (Mantel-Cox) tests was used to analyze significant difference between survival curves.

## Results

### DMXAA has adjuvant properties and induces preferential type-2 response

**Figure 1:**
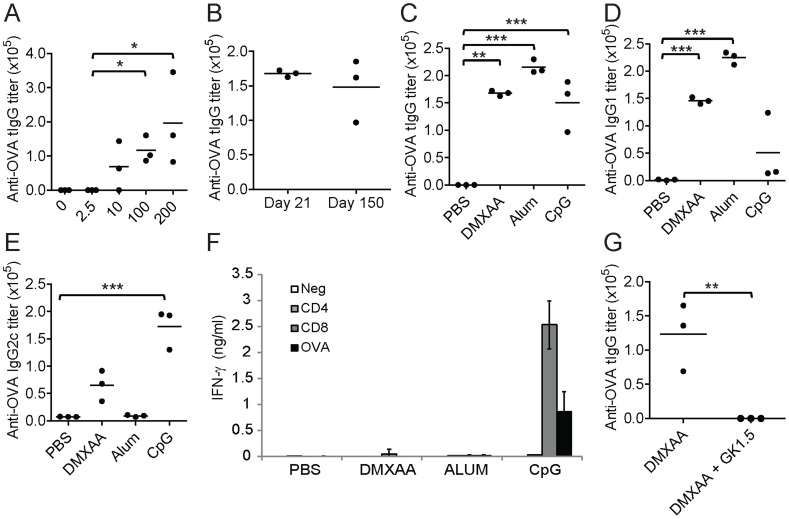
DMXAA acts as a potent adjuvant. (A) Anti-OVA tIgG titers of C57BL/6 mice immunized with 100 µg OVA plus the indicated doses of DMXAA (µg). (B) Anti-OVA tIgG titers of C57BL/6 mice 21 days and 150 days after immunization with 100 µg OVA and 100 µg DMXAA. (C–E) C57BL/6 mice were immunized twice i.d. with 100 µg OVA plus DMXAA (100 µg), Alum (665 µg) or CpG DNA (25 µg) and the induction of (C) tIgG, (D) IgG1 and (E) IgG2c antibody responses against OVA were assessed. (F) IFN-γ secretion from splenocytes of immunized mice that were stimulated for 48 h with CD4 and CD8 OVA peptides and whole OVA protein. (G) Anti-OVA tIgG titers of C57BL/6 mice injected i.v. with 200 µg anti-CD4 (GK1.5) antibodies prior to immunization with 100 µg OVA and 100 µg DMXAA. Results presented are representatives of three separate experiments. *<0.05, ** *P*<0.01, *** *P*<0.001 by Students t-test when comparing between two groups and one-way ANOVA with Bonferroni's post-test when comparing three or more groups.

To determine if the immunogenic property of DMXAA could adjuvant vaccines, we utilized the OVA model antigen system where C57BL/6 mice were immunized with OVA mixed with DMXAA. We found that DMXAA could significantly augment specific immune responses against OVA, as indicated by the increase in serum anti-OVA total IgG (tIgG) titers compared to OVA alone immunized group (Figure 1A). The adjuvant effect was dependent on the dose of DMXAA. In addition, it was observed to have noticeable but insignificant adjuvant effect at a low dose of 10 µg. The immune response induced by the combination of DMXAA and OVA was long-lasting and could be detected as late as 150 days after the final immunization (Figure 1B). To evaluate its efficacy, we compared DMXAA with the established adjuvants, Alum and CpG DNA, which induce predominantly T_H_2 and T_H_1 immune responses, respectively. Mice immunized with OVA plus DMXAA (100 µg) generated comparable anti-OVA tIgG titers as Alum (665 µg) and CpG DNA (25 µg) adjuvanted groups (Figure 1C). DMXAA resembled Alum in generating predominantly T_H_2 type responses as indicated by the induction of higher IgG1 than IgG2c titers (Figure 1D and 1E). In contrast, CpG DNA induced higher IgG2c and lower IgG1 levels. We have also analyzed OVA specific T cell responses by stimulating splenocytes of immunized mice with whole OVA protein or its CD4 and CD8 epitopes followed by measuring IFN-γ secretion. No T-cell responses could be detected in OVA plus Alum or DMXAA groups, whereas splenocytes from the OVA plus CpG group responded with high IFN-γ secretion in the presence CD8 peptide and whole OVA protein (Figure 1F). *In-vivo* depletion of CD4 T cells prior to immunization with OVA and DMXAA completely abrogated the production of OVA-specific antibodies (Figure 1G), suggesting that the generation of adaptive immune responses by DMXAA was CD4 T cell-dependent. These results indicate that DMXAA possesses immuno-stimulatory properties that can function effectively as an adjuvant for vaccines.

### Adjuvant effect of DMXAA is dependent on the type-I-IFN response induced by IRF3 signaling

DMXAA has been shown to activate the TBK1-IRF-3 signaling pathway to induce strong IFNβ response from mouse embryonic fibroblasts (MEFs), macrophages and dendritic cells [7]. A recent study also reported that DMXAA could induce IL-33 up-regulation through IRF3 dependent mechanism [22]. IL-33 promotes humoral immunity by triggering the release of T_H_2 cytokines such as IL-4, IL-5 and IL-13 from polarised naive T cells [23]. Therefore we would like to determine if the adjuvant effect of DMXAA requires IRF3-dependent type-I-IFN secretion and could the induction of preferential T_H_2 type response be due to its up-regulation of IL-33.

**Figure 2:**
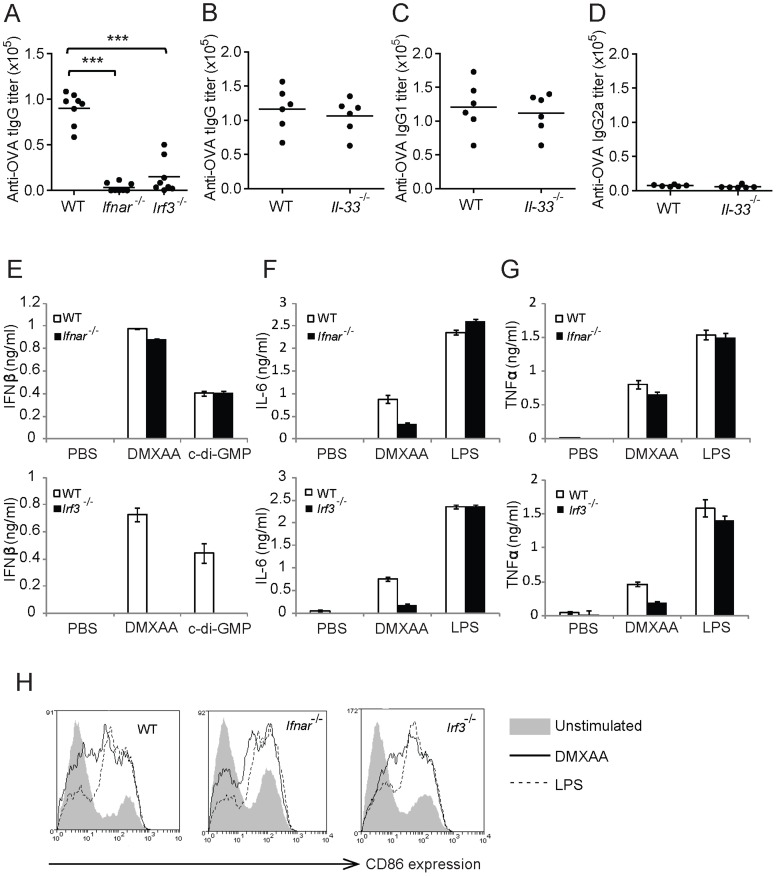
Adjuvant effects of DMXAA require type-I-IFN responses induced by IRF3 activation. Anti-OVA (A) tIgG antibody responses of WT C57BL/6, *Ifnar^-/-^* and *Irf3^-/-^* mice immunized twice i.d. with 10 µg OVA plus 100 µg DMXAA. (B) tIgG, (C) IgG1 and (D) IgG2a antibody titers against OVA in WT BALB/c and *Il-33^-/-^* mice immunized twice i.d. with 10 µg OVA plus 100 µg DMXAA. Results presented are pooled titers from two separate experiments. *In-vitro* cultured DCs derived from WT, *Ifnar^-/-^* and *Irf3^-/-^* mice were stimulated with DMXAA (2.5 µg/ml), LPS (1 µg/ml) or lipofectamine complexed c-di-GMP (10 µg/ml) for 6 h before the supernatant were collected and analysed for (E) IFNβ, (F) IL-6 and (G) TNFα secretion and CD11c^+^ cells were analysed for CD86 expression (H). Results presented are average of triplicate conditions ± SD and are representative of three separate experiments. *** *P*<0.001 one-way ANOVA with Bonferroni's post-test.

To address this, OVA immunization studies were performed on mice lacking IRF3 (*Irf3* ^-/-^), IFNαβ receptor (*Ifnar* ^-/-^) and IL-33 (*Il-33* ^-/-^). As observed in Figure 2A, the anti-OVA tIgG (Figure 2A) titers from *Irf3* ^-/-^ and *Ifnar* ^-/-^ mice were significantly inhibited compared to WT C57BL/6 mice. This indicates that the adjuvant effect of DMXAA was strongly dependent on IRF3 mediated transcription and responses mediated by type-I-IFN. In contrast to *Irf3^-/-^* and *Ifnar* ^-/-^ mice, *Il-33* ^-/-^ mice showed comparable levels of tIgG antibody response as WT BALB/c immunized mice (Figure 2B). Moreover, the preference for the induction of IgG1 (Figure 2C) over IgG2a (Figure 2D) subtype as observed in WT BALB/c mice remained the same in *Il-33* ^-/-^ mice. To further support the dependence on IRF3 mediated type-I-IFN for DMXAA adjuvant effect, bone marrow derived DCs from *Irf3* ^-/-^, *Ifnar* ^-/-^and WT mice were stimulated with DMXAA (Figure 2E–G). Cyclic diguanylate (c-di-GMP) is an IRF3-dependent type-I-IFN inducer and was included as a control. As observed in figure 2E, *Irf3* ^-/-^ DCs were unable to induce IFNβ response whilst *Ifnar* ^-/-^ DC responded with levels comparable to WT DCs. Therefore indicating that the lack of DMXAA adjuvant effect observed in *Ifnar* ^-/-^ mice was not due to the inability to induce type-I-IFN but rather it was the inability to respond to it. Although *Irf3* ^-/-^ and *Ifnar* ^-/-^ mice did not respond to the adjuvant effect of DMXAA, it was found to be capable of inducing IL-6 (Figure 2F) and TNFα (Figure 2G) response from *Irf3* ^-/-^ and *Ifnar^-/-^* DCs. In addition, the DC maturation effect of DMXAA was still present in *Irf3^-/-^* and *Ifnar^-/-^* DCs in the same order of magnitude as WT DCs (Figure 2H). These data suggest that other stimulatory pathways of DMXAA remained intact in *Irf3^-/-^* mice but they did not play a role in the adjuvant effects of DMXAA. Collectively, we demonstrate that the adjuvant effect of DMXAA is directly dependent on IRF3 mediated type-I-IFN induction and that the reported IL-33 up-regulation by DMXAA is not involved in raising immunogenicity of the vaccine or the skewing towards Th2 type response.

### DMXAA is a potent adjuvant for influenza split virus vaccine and enhances protection against influenza challenge

**Figure 3:**
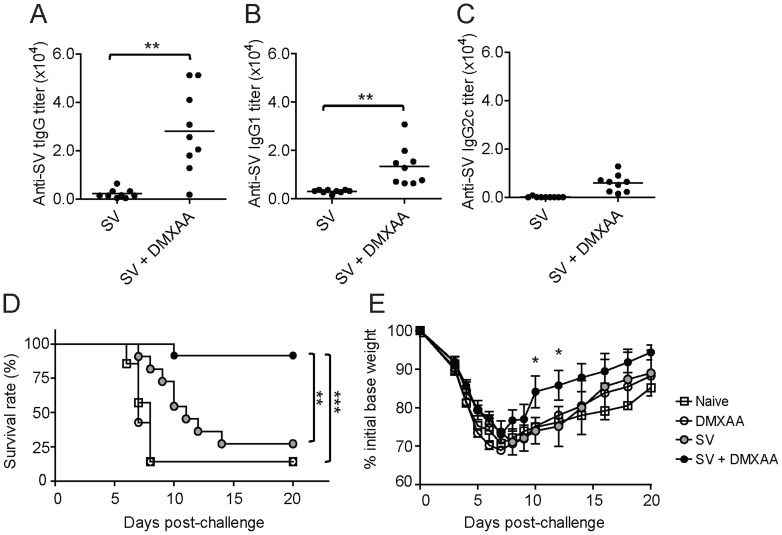
Adjuvant effect of DMXAA can improve potency of influenza SV vaccine and protect mice from a lethal challenge. C57BL/6 mice were immunized twice i.d. with 0.75 µg SV and 100 µg DMXAA, and their sera were assessed for anti-SV (A) tIgG, (B) IgG1 and (C) IgG2c antibodies. ** *P*<0.01 by Students t-test. Results presented are pooled from two separate experiments. (D) C57BL/6 mice that had received two i.d. injections of PBS (open square), 100 µg DMXAA alone (open circle), 0.75 µg SV alone (grey-filled circle) or SV plus DMXAA (black filled circle) were challenged with a lethal dose of A/Puerto Rico/8/34 (PR) (H1N1) 7 days after the final immunization (n = 6 mice per experimental group). Results presented are pooled from two separate experiments. Survival rates were recorded daily and statistical analyses were performed using the log-rank (Mantel-Cox) test where ** and *** denotes p<0.01 and p<0.001 respectively (E) The rate of weight-loss by the challenged mice were monitored and presented as an average percentage of the initial base weight ± standard error. * denotes p<0.05 vs SV alone by Student's T-test.

In our previous report, we have demonstrated that in contrast to influenza whole virus vaccine (WV), split vaccine (SV) was unable to induce type-I-IFN production from plasmacytoid DCs [21]. This was due to the lack of RNA content in the SV preparation required to trigger TLR7 activation. As a result, SV immunizations were less protective against lethal influenza challenge as compared to WV immunizations. Hence, we would like to determine if the type-I-IFN dependent adjuvant effect of DMXAA could adjuvant SV and immunized mice from live flu challenges. C57BL/6 WT mice were immunized intradermally with SV prepared from New Caledonia/20/1999 (H1N1) and mixed with DMXAA. We found that SV plus DMXAA induced higher tIgG antibody responses than SV alone immunizations (Figure 3A). Similar to OVA immunization studies, the adjuvant effect of DMXAA induced higher IgG1 than IgG2c titers to SV (Figure 3B and 3C). Next, we challenged the immunized mice with a high dose of A/Puerto Rico/8/34 (PR) (H1N1). As seen in Figure 3D, naive mice were quick to succumb to the selected dose of live influenza challenge whilst SV alone immunized mice were offered low level protection (Figure 3A). Although SV alone immunizations had low antibody titers, it was found to be mildly immunogenic and capable of inducing detectable CXCL10 and Anti-HA BALF IgA production [21] that may account for the low level of protective response observed. In contrast, mice immunized with SV plus DMXAA had significantly higher survival rates than naive mice and mice immunized with SV alone (Figure 3D). 11 out of 12 mice that were immunized with SV + DMXAA survived the lethal challenge compared to the 3 out of 12 SV alone group. It was also observable from the rate of weight-loss that SV plus DMXAA immunized mice had a lesser degree of disease-induced morbidity and were able to recover from the infection at a faster rate than control groups (Figure 3E). To exclude the possible role of DMXAA-induced innate immune responses in the protection against lethal challenge, the survival rate of mice injected with DMXAA alone without SV was determined and found to be similar as naïve mice (Figure 3D). Therefore the protective response observed in SV + DMXAA immunized group was due to the adaptive response generated from the immunization and not the innate immune response triggered by DMXAA. These results demonstrate that DMXAA is an efficacious adjuvant for SV vaccine.

## Discussion

A large cohort Phase III clinical trial of DMXAA on patients with non-small cell lung carcinoma was recently halted due to inefficacy although it was shown to be well tolerated [1]. As opposed to an earlier successful Phase II clinical trial [24], the Phase III trial showed no overall survival between DMXAA and placebo treated groups. The researchers conducting the clinical trial reasoned that a smaller sample size in the phase II trial overestimated the efficacy of DMXAA. The future of DMXAA as a vascular disruptive agent for cancer therapy is therefore uncertain. In this report, we have demonstrated that the immunogenic properties of DMXAA could be harnessed to adjuvant vaccines with its acceptable safety profile. A local low-dose of DMXAA was capable of adjuvanting vaccines with efficacy that was comparable to the well-studied adjuvants, Alum and CpG. The adjuvant activity was observed using amounts as low as 10 µg per mouse, which was a smaller dose than the 30 mg/kg required for the vascular disruptive effect [25]. When extrapolated to human use, the lower dose required for the adjuvant activity serves to promote DMXAA as a candidate for vaccine adjuvant.

Despite the activation of several distinct inflammatory signaling pathways, we narrowed the immune activity responsible for the adjuvant effect of DMXAA to the IRF3 mediated activation of type-I-IFN. This is surprising as DMXAA induced biased T_H_2 response while type-I-IFN is commonly associated with the generation of T_H_1 response *in-vivo* [26]. The recent study reporting that DMXAA could induce IL-33 up-regulation through IRF3 dependent mechanism made us question if this could be the reason for the unusual observation [22]. However, immunization studies performed on *Il-33^-/-^* mice confirmed that IL-33 was not involved in the adjuvant effect of DMXAA or its skewing towards T_H_2 response. We have recently reported that Alum mediates enhancement of T_H_2 response through the DNA sensing pathway triggered by the release of dsDNA from dying host cells [18]. However, we found that DMXAA did not induce significant increase in free dsDNA in the peritoneal lavage of mice when injected intraperitoneally as opposed to Alum (data not shown). Therefore the mechanism through which DMXAA induced preferential T_H_2 type responses remains elusive and requires further investigation. It is possible that the production of IL-6 by DMXAA to be involved as it has been known to inhibit T_H_1 polarization by activating NFAT, c-maf and SOCS-1 [27], [28] and induce the humoral immunity promoting cytokine, IL-21.

The revealing of DMXAA adjuvant property suggests that it could adjuvant tumor associated antigens and activate the adaptive immune system against cancer cells as part of its anti-tumor response. So far, there are no reports on DMXAA raising humoral immunity against tumor cells with its T_H_2 enhancing capability. However, there is evidence which suggests that DMXAA could act as a cancer vaccine adjuvant. For example, it was demonstrated that the administration of DMXAA in tumor bearing mice could increase the number of circulating specific CD8 T-cells [16]. It was also shown to have a positive influence in a separate study which investigated if the anti-cancer property of systemic high-dose DMXAA could work in combination with the adaptive immune response generated by DNA vaccine to protect mice against tumor challenges [29].

In summary, results from this report have shown that DMXAA is capable of functioning as an adjuvant with a defined mechanism that acts specifically on the IRF3 dependent induction of type-I-IFN. DMXAA has already been investigated for applications in antiviral [15] and anti-bacterial [30] therapies and here we demonstrate that it is capable of adjuvanting vaccines as well.
